# Our current understanding of the toxicity of altered mito-ribosomal fidelity during mitochondrial protein synthesis: What can it tell us about human disease?

**DOI:** 10.3389/fphys.2023.1082953

**Published:** 2023-06-30

**Authors:** Antón Vila-Sanjurjo, Natalia Mallo, John F. Atkins, Joanna L. Elson, Paul M. Smith

**Affiliations:** ^1^ Grupo GIBE, Departamento de Bioloxía e Centro de Investigacións Científicas Avanzadas (CICA), Universidade da Coruña (UDC), A Coruña, Spain; ^2^ Schools of Biochemistry and Microbiology, University College Cork, Cork, Ireland; ^3^ The Bioscience Institute, Newcastle University, Newcastle uponTyne, United Kingdom; ^4^ Human Metabolomics, North-West University, Potchefstroom, South Africa; ^5^ Department of Paediatrics, Raigmore Hospital, Inverness, Scotland, United Kingdom

**Keywords:** mito-ribosome, mtDNA, mitochondrial rRNA mutations, translational fidelity, mtDNA diseases, deafness (hearing loss), proteostasis, longevity

## Abstract

Altered mito-ribosomal fidelity is an important and insufficiently understood causative agent of mitochondrial dysfunction. Its pathogenic effects are particularly well-known in the case of mitochondrially induced deafness, due to the existence of the, so called, ototoxic variants at positions 847C (m.1494C) and 908A (m.1555A) of 12S mitochondrial (mt-) rRNA. It was shown long ago that the deleterious effects of these variants could remain dormant until an external stimulus triggered their pathogenicity. Yet, the link from the fidelity defect at the mito-ribosomal level to its phenotypic manifestation remained obscure. Recent work with fidelity-impaired mito-ribosomes, carrying error-prone and hyper-accurate mutations in mito-ribosomal proteins, have started to reveal the complexities of the phenotypic manifestation of mito-ribosomal fidelity defects, leading to a new understanding of mtDNA disease. While much needs to be done to arrive to a clear picture of how defects at the level of mito-ribosomal translation eventually result in the complex patterns of disease observed in patients, the current evidence indicates that altered mito-ribosome function, even at very low levels, may become highly pathogenic. The aims of this review are three-fold. First, we compare the molecular details associated with mito-ribosomal fidelity to those of general ribosomal fidelity. Second, we gather information on the cellular and organismal phenotypes associated with defective translational fidelity in order to provide the necessary grounds for an understanding of the phenotypic manifestation of defective mito-ribosomal fidelity. Finally, the results of recent experiments directly tackling mito-ribosomal fidelity are reviewed and future paths of investigation are discussed.

## Altered mito-ribosomal fidelity is an important and insufficiently understood causative agent of mitochondrial dysfunction

The importance of mitochondrial DNA (mtDNA) disease in global human health is now widely recognized, with a prevalence of 9.6 cases per 100,000 individuals (Craven et al., 2017). Mitochondrial diseases are clinically heterogeneous and usually display high tissue specificity, commonly affecting post-mitotic tissues with high energy demand, such as skeleton muscle, cochlea, heart, or brain (Craven et al., 2017). An additional layer of complexity has been demonstrated by studies in flies and mice showing that diet can also affect the phenotypic manifestation of mtDNA mutations (Aw et al., 2018; Ferreira et al., 2019; Richman et al., 2021).

Given the central role of the mito-ribosome in mt-gene expression, the fact that some mt-rRNA mutations have been proposed as causative agents of mtDNA disease is not surprising. However, much less is known about disease causing mutations in the mt-rRNA genes than in any of the other mtDNA genes, largely because mt-translation has remained highly refractory to direct measurements. In addition, despite the existence of important conservation of rRNA structure between bacterial and mitochondrial ribosomes, the special evolution of the latter led to the adoption of novel rRNA folding solutions in many regions of the particle. As a result, nucleotides mapping to these regions bear a minor-to-null phylogenetic imprint that can hardly be used in comparative studies to infer their structural and/or functional importance (Vila-Sanjurjo et al., 2021).

Cases of deafness, myopathy, respiratory deficiencies, Leigh’s disease, optic atrophy, congenital glaucoma, Dupuytren’s disease, MELAS, and other disorders have been linked to potentially pathogenic mt-rRNA mutations (Smith et al., 2014; Elson et al., 2015a; Elson et al., 2015b). In addition, several mt-rRNA mutations with high disruptive potential have been identified in association with cancerous processes (Smith et al., 2014; Elson et al., 2015a; Elson et al., 2015b; Haumann et al., 2020). The case of the deafness-inducing variants is particularly intriguing, with more than 80 suspected variants whose pathogenic role remains uncertain (Vila-Sanjurjo et al., 2023). Despite this apparent abundance, the two known ototoxic mutations of mt-12S rRNA, 847C>A (m.1494C>T) and 908A>G (m.1555A>G) (in keeping with our previous nomenclature for mtDNA variants (Smith et al., 2014; Elson et al., 2015a; Elson et al., 2015b), we provide the gene numbering first, followed by genome numbering), remain as the only mt-rRNA variants with proven pathogenicity, with a prevalence in the European population estimated to be higher than 1 in 500 adults (Prezant et al., 1993; Vandebona et al., 2009; Zhao et al., 2004). Studies performed in bacteria indicated that mutations at the rRNA residues equivalent to m.1494C and m.1555A acted by decreasing the accuracy of decoding during protein synthesis, *i.e*., by decreasing the fidelity of mt-translation. The toxic effect of aminoglycoside antibiotics (AGs), a subset of antibiotics with a well-known role in altering translational fidelity, is a key element in determining the penetrance of these variants. AGs are some of the most commonly prescribed antibiotics, despite their well-known capacity to cause toxic side effects to the kidneys and inner ear (Huth et al., 2011). While their use in the industrialized world is usually limited to severe infections, they are very popular in the developing world due to their low cost and potent antibacterial activities (Huth et al., 2011). Beyond their anti-bacterial use, the potential of certain aminoglycosides to ameliorate the symptoms of genetic diseases caused by mutations resulting in premature stop codons, has been extensively explored (Bidou et al., 2012). The high prevalence of mitochondrial deafness, together with the extensive anti-bacterial use of AGs, makes it urgent to elucidate the role of the mito-ribosome in pathogenesis, more specifically, how altered levels of mito-ribosomal fidelity affect mitochondrial and cellular activities, finally leading to disease.

New research is changing the way in which we look at the mito-ribosome and mito-ribosomal fidelity. In particular, new high-resolution structures of the mito-ribosome are bringing an unprecedented view of the architecture of this molecular machine, underscoring the similarities, but also the radical differences existing between this particle and its counterparts across the phylogenetic spectrum (Kummer and Ban., 2021). Our understanding of how translational fidelity is amplified into a cellular and even organismal response, mainly *via* its interference with altered proteostasis, has increased enormously through evidence obtained in non-mitochondrial systems. This type of research provides a framework in which the effects of translational accuracy can be understood beyond its ribosomal origin. Finally, several examples exist now in which mito-ribosomal fidelity has been purposely manipulated. Such examples constitute the first direct link between mito-ribosomal accuracy and pathogenesis. This review attempts to put all this evidence into context, so that the reader will be able to grasp the, so far, under-appreciated role of mito-ribosomal fidelity in human disease with the hope that more patients and mutations can be identified allowing for the provision of improved patient support.

## The ribosome and the fidelity of translation

The ribosome is currently understood as an active and dynamic hub for protein and mRNA quality control (Pechmann et al., 2013; D'Orazio and Green., 2021). To achieve this, ribosomes act at several levels including, recognition of mRNA during initiation; threading of the mRNA at the ribosomal codon recognition sites; promoting tRNA-induced conformational changes to facilitate codon-anticodon recognition and proofreading during elongation; sensing the presence of damaged mRNAs; detecting and degrading stalled nascent protein chains; detecting non-cognate tRNAs after peptidyl transfer to avoid amino acid misincorporation; and sensing the nature of the nascent protein chain at the exit ribosomal tunnel (Janssen and Hayes., 2012; Pechmann et al., 2013; Sitron and Brandman., 2020). Mitochondrial ribosomes are among those with quality control mechanisms. In particular, two factors belonging to the release factor family, namely, ICT1 (mL62), and C12orf65 (mtRF-R), are specialized in rescuing stalled mito-ribosomes, constituting part of the mitochondrial machinery that is responsible for co-translational ribosome-associated quality control or RQC (Desai et al., 2020; Kummer et al., 2021). While we are well in our way to ascertain the apparent sophistication of mito-ribosomes, tackling the fidelity of mitochondrial translation has been an intractable issue until very recently.

**FIGURE 1:**
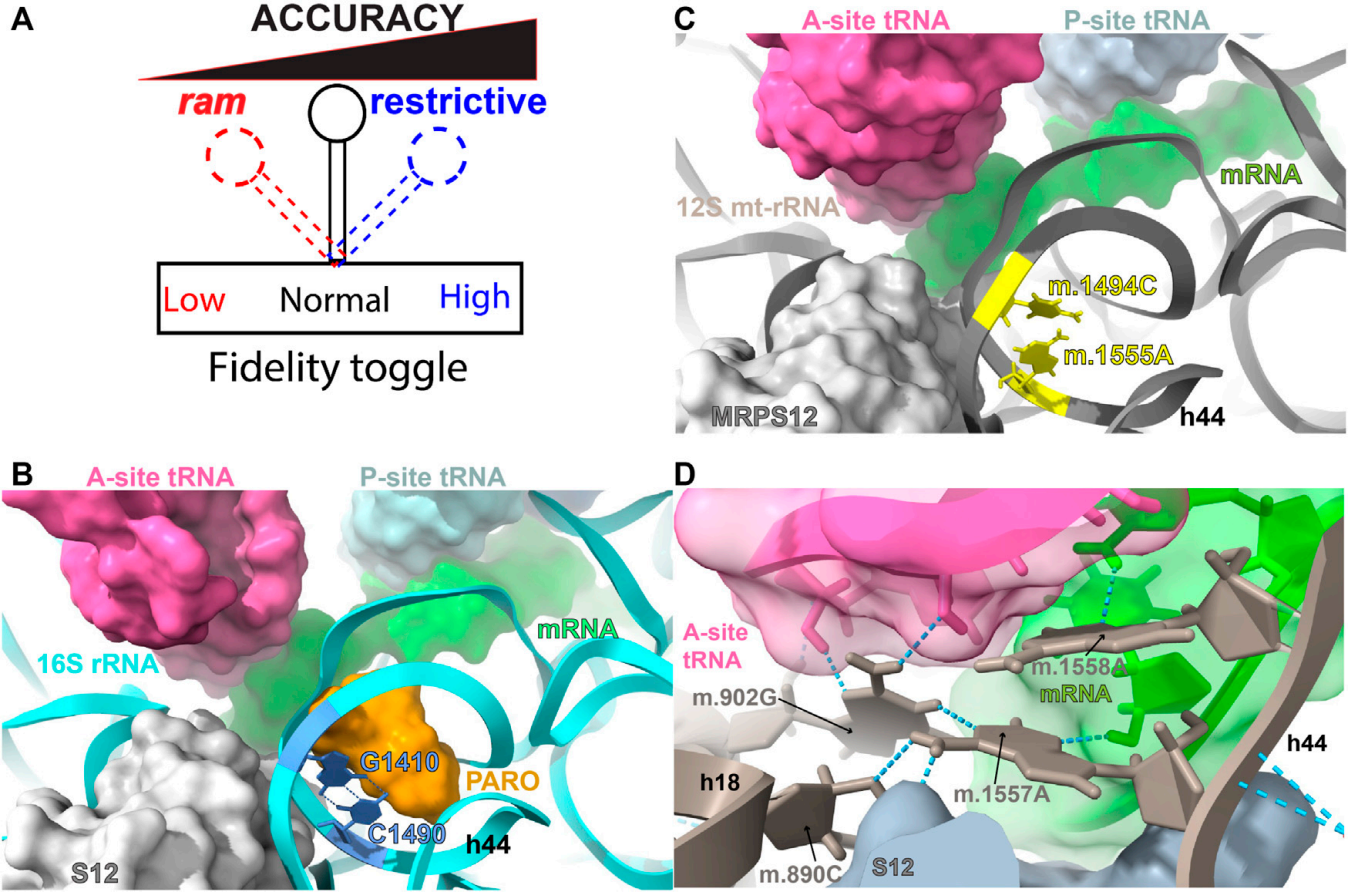
DC and AG binding sites. **(A)** The ribosomal fidelity toggle. **(B)** Bacterial decoding center (DC) and aminoglycoside (AG) binding site. **(C)** Human mitochondrial DC and AG binding site. **(D)** Hydrogen bonding network during codon-anticodon recognition. The location of bacterial residues G1410 and C1490 (blue) of 16S rRNA (*T. thermophilus*) and 847C (m.1494C) and 908A (m.1555A) (yellow) of 12S mt-rRNA are shown in **(B,C)**. Hydrogen bonds between G1410 and C1490 are indicated by blue, broken lines in **(B)**. The aminoglycoside antibiotic paromomycin (orange), present in the bacterial structure, is shown in orange in **B**. Other important molecules present in the decoding center (A-, P-site tRNA, mRNA, and r-protein S12/MRPS12/uS12m) are shown in **(B,C)**. Methods: To create panels **(A–C)**, the 2.2-Å cryo-EM human mito-ribosomal structure (Itoh et al., 2021; Itoh et al., 2022) and the structure of the *T. thermophilus* 70S ribosome complexed with mRNA, tRNA and (PDB Accession number: 4YBB) were superposed with the Matchmaker utility of Chimera X (Pettersen et al., 2021), using protein S12 as a reference chain.

Though the error rate is sequence specific and influenced by several factors, the composite error rate during protein synthesis is approximately one mis-incorporated amino acid once every 10^3^–10^4^ codons, resulting in around 18% of all translated proteins containing a single error (Zaher and Green., 2009; Taylor and Dillin., 2011). Early studies showing the weakness of the codon-anticodon interaction in solution (Lipsett et al., 1960), indicated that this interaction, by itself, could not achieve the level of discrimination rates observed in translation. The discovery of the misreading effect of streptomycin, followed by the isolation of a conditional streptomycin-dependent, auxotrophic mutant of *Escherichia coli* by Gorini and co-workers, led to the conclusion that the ribosome must play a fundamental role in the stabilization of the codon-anticodon interaction during protein synthesis and that ribosomal structure influenced the accuracy of genetic decoding (Spotts and Stanier, 1961; Davies et al., 1964; Gorini and Kataja, 1964; McLaughlin et al., 1966; Birge and Kurland., 1969). Additional streptomycin-related mutations were rapidly discovered, leading to the creation of the following two categories of fidelity mutations. On the one hand, restrictive or hyper accurate mutations, composed of streptomycin-resistant, -dependent, and -pseudo-dependent mutations that mapped to ribosomal protein S12 (Ozaki et al., 1969; Birge and Kurland., 1970; Nomura, 1970; Zengel et al., 1977). Restrictive mutations were found to reduce the error rate of the ribosome. On the other hand, *ram* or *r*ibosomal *am*biguity mutations, mapping to ribosomal proteins S4 and S5, which were capable of suppressing the streptomycin-dependent phenotype by causing extensive miscoding (Rosset and Gorini., 1969; Nomura, 1970). Taken together, these data clearly demonstrated that the ribosome controlled the fidelity of genetic decoding and that a ribosomal “fidelity toggle” (Figure 1A) existed that could be shifted towards more or less decoding accuracy. Given that the bacterial ribosome is mainly composed of rRNA, additional fidelity mutations, including some that affected framing, were eventually found in the rRNA component of the ribosome (O’Connor and Dahlberg., 1995; O’Connor et al., 1997; Liiv and O'Connor., 2006; O'Connor., 2007; McClory et al., 2010; Sun et al., 2011; Tran et al., 2011; Hoffer et al., 2019).

All the above means that some translational fidelity defining events, such as stop codon readthrough and frameshifting are an intrinsic property of the ribosome. This was not fully appreciated early on, especially in the case of frameshifting. By the time the deciphering of the genetic code was completed and even afterwards, it was thought that infidelity at the transcription level, due to untemplated insertions or deletions, and infidelity at the translational level due to ribosomal frameshifting, would be so deleterious that natural selection would have ensured that neither occurred. How could proteins, especially giant proteins, otherwise be synthesized? Further, it was assumed that no single mutational change in a gene for a transcriptional or translational component, or a template context feature, would allow even localized changes of reading frame different from those specified by the start codon. At the time, it was thought that frameshift mutations (caused by a base insertion or deletion due to RNA polymerase slippage) could not be “leaky,” in the sense of being capable of producing some amount of full length product (Newton, 1970). Furthermore, frameshift mutations could not be compensated for or suppressed by, a change in a different gene (Whitfield et al., 1966). However, in a collaborative effort employing the ribosome fidelity mutants of the Gorini lab, we were able to identify ribosomal frameshifting events that were distinct from any transcriptional errors due to RNA polymerase slippage (Atkins et al., 1972; Atkins et al., 1979). Today we know that ribosomal frameshifting is quite common and is considered an important mechanisms of regulation of gene expression (Atkins et al., 2016). The use of frameshifting in the mitochondrial context has been proposed as a mechanism to allow the termination of translation on the mammalian *MTCO1* and *MTND6* ORFs (Temperley et al., 2010). Despite the beauty of this mechanism, whether it actually occurs has been called into question (Kummer and Ban., 2021).

The physical separation between the decoding center (DC) of the ribosome, where codon-anticodon recognition occurs, and the peptidyl transferase center (PTC), where amino acids polymerize, is ∼70 Å. This distance, together with the fact that both reactions occur in different ribosomal subunits (SSU vs. LSU, respectively), imposes a problem related to the temporal and physical coupling of the two events. Most of the studies, especially the early studies regarding the molecular nature of ribosomal decoding were performed in bacteria. More recent studies of yeast and mammalian 80S ribosomes, including those on accuracy by Djumagulov et al. (2021), are also providing deep structural insights. Comparison of the recent high-resolution structures of the mammalian mito-ribosome to other available ribosomal structures has confirmed that the three-dimensional structure all ribosomal DCs is highly conserved across the phylogenetic spectrum (Figures 1B, C) (Amunts et al., 2015; Greber et al., 2015). Thus, many of the lessons learned about ribosomal decoding in bacteria and other model organisms are applicable to the mitochondrion. Despite this and other structural similarities in conserved regions, bacterial and mitochondrial ribosomes are quite distinct, with a reversed RNA:protein ratio (2:1 vs. 1:2) that leads to drastic structural structural differences in regions beyond the main functional centers (Amunts et al., 2015; Greber et al., 2015).

The DC maps to the SSU at the interface between this subunit and the LSU. The DC is composed of several structurally conserved helices of the SSU rRNA, carrying a few universally conserved residues that are involved in monitoring the geometry of the codon-anticodon interaction at the A site (for an exhaustive review of ribosomal fidelity, see Zaher and Green., 2009). In particular, three such residues undergo a drastic conformational change that is responsible for the induced-fit mechanism used during decoding, to discriminate between cognate and non-cognate tRNAs in the SSU (Pape et al., 1999; Ogle et al., 2001; Geggier et al., 2010). In the human mito-ribosome these residues are positions 255G (mt.902G)^1^ in helix h18 of 12S mt-rRNA (equivalent to the bacterial G530) and the two tandem adenosines 910A (m.1557A) and 911A (m.1558A) in h44 (equivalent to the bacterial A1492 and A1493) (Carter et al., 2000; Ogle et al., 2001; Ogle et al., 2002; Demeshkina et al., 2012; Itoh et al., 2021; Itoh et al., 2022). Figure 1D shows how these residues establish a hydrogen bonding network with the mRNA and tRNA residues involved in the formation of a cognate codon-anticodon interaction in the mito-ribosomal A site. The structure shows that the base of 910A (m.1557A) establishes hydrogen bonds with residues 243C (m.890C) and 255G (m.902G) of h18 (equivalent to bacterial C518 and C530, respectively) and with the mRNA ribose at position +5 (+1 is the first position of the P-site codon). In contrast, position 911A (m.1558A) uses its base to establish hydrogen bonds with the O2’ at position 36 in the anticodon stem loop of A-site tRNA and with the mRNA base at position +4, while establishing an additional hydrogen bond with position +4 *via* its O2′ group (Figure 1D). Such an interaction is almost identical with the one described in bacteria, except for the primary sequence of the codon and anticodon (Ogle et al., 2001; Demeshkina et al., 2012; Itoh et al., 2021; Itoh et al., 2022). The structure also shows direct contacts between MRPS12/uS12m with the base and rRNA backbone at position 910A (m.1557A), attesting to the influence of this protein in mito-ribosomal decoding (Figure 1D) (Ogle et al., 2001; Itoh et al., 2021; Itoh et al., 2022).

**FIGURE 2:**
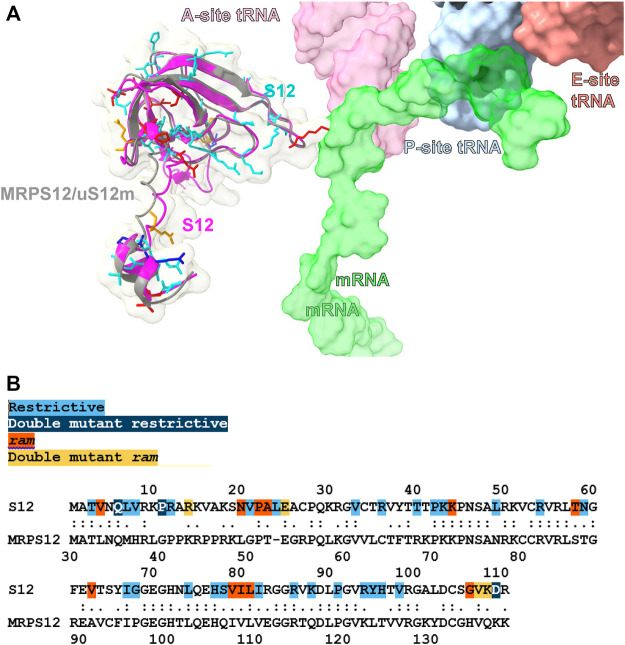
Structural comparison of the bacterial and human mitochondrial versions of ribosomal protein S12. **(A)**. Superposition of the bacterial S12 (magenta) and human mitochondrial MRPS12/uS12m (gray) proteins. Bacterial sites of fidelity mutations are color coded as indicated in **(B)** and shown in stick representation. MRNA, E-, P-, and A-site mt-tRNAs are shown in surface representation (Itoh et al., 2021; Itoh et al., 2022). **(B)** Alignment between the *Escherichia coli* S12 and human mitochondrial MRPS12/uS12m proteins. Sites of S12 fidelity mutations are color coded as indicated (Gregory et al., 2001; Agarwal et al., 2011; Datta et al., 2021). Methods: To create panels **(A, B)**, the 2.2-Å cryo-EM human mito-ribosomal structure (Itoh et al., 2021; Itoh et al., 2022) and the structure of the *E. coli* 70S ribosome complexed with mRNA, tRNA and (PDB Accession number: 4YBB) were superposed with the Matchmaker utility of Chimera X (Pettersen et al., 2021), using protein S12 as a reference chain.

Besides induced fit, codon–anticodon recognition upon tRNA binding at the A site triggers the so called open-closed transition of the SSU in which the subunit undergoes a long-range conformational change that drives tRNA selection in the forward direction (Ogle et al., 2001; Ogle et al., 2002; Demeshkina et al., 2012). Bacterial ribosomal protein S12 is a crucial part of this transition, a fact that was used to explain the phenotypic effect of the classic fidelity mutations mapping to this protein as well as to proteins S4 and S5 (Ogle et al., 2001; Ogle et al., 2002). The intimate relationship between the structure of S12 (and its orthologues) in decoding was further underscored by Agarwal et al. (Agarwal et al., 2011), who uncovered the existence of many additional S12 fidelity mutations, sparsely distributed throughout its primary sequence (Figures 2A, B). This finding provided a clear view of the sensitivity of ribosomal fidelity to subtle changes in the structure of S12. While the open-closed transition could partly explain the phenotypic effects of these mutations, structural analysis of S12 mutants has led to a complementary view in which, prior to domain closure, the protein directly pre-organizes the open state of the decoding site in preparation for A-site substrate binding and codon recognition (Demirci et al., 2013). In this view, protein S12 no longer plays a simple bystander role in the decoding process but instead contributes directly to the dynamics of codon-anticodon recognition, by structuring the decoding site during the early stages of the decoding process (Demirci et al., 2013).

MRPS12/uS12m is an early-binding mito-ribosomal protein that is structurally almost identical to its bacterial counterpart (Figure 2A) (Amunts et al., 2015; Greber et al., 2015; Sanchez et al., 2021). Hence, it is expected that the function of this protein in the mito-ribosome is also conserved. Besides the structural evidence, the information on the role of MRPS12/uS12m in mito-ribosomal function is scarce. The importance of protein MRPS12/uS12m in mito-ribosomal function was first revealed by the finding that a missense mutation in the *Drosophila* MRPS12/uS12m resulted in lower amounts of mito-ribosomes and decreased mitochondrial translation (Toivonen et al., 2001). More recently, proof of the implication of MRPS12/uS12m in mammalian translational fidelity has been obtained by the recent generation of MRPS12/uS12m fidelity mutants in mice (see below) (Ferreira et al., 2019).

## The fidelity of mito-ribosomal translation and human disease

**FIGURE 3:**
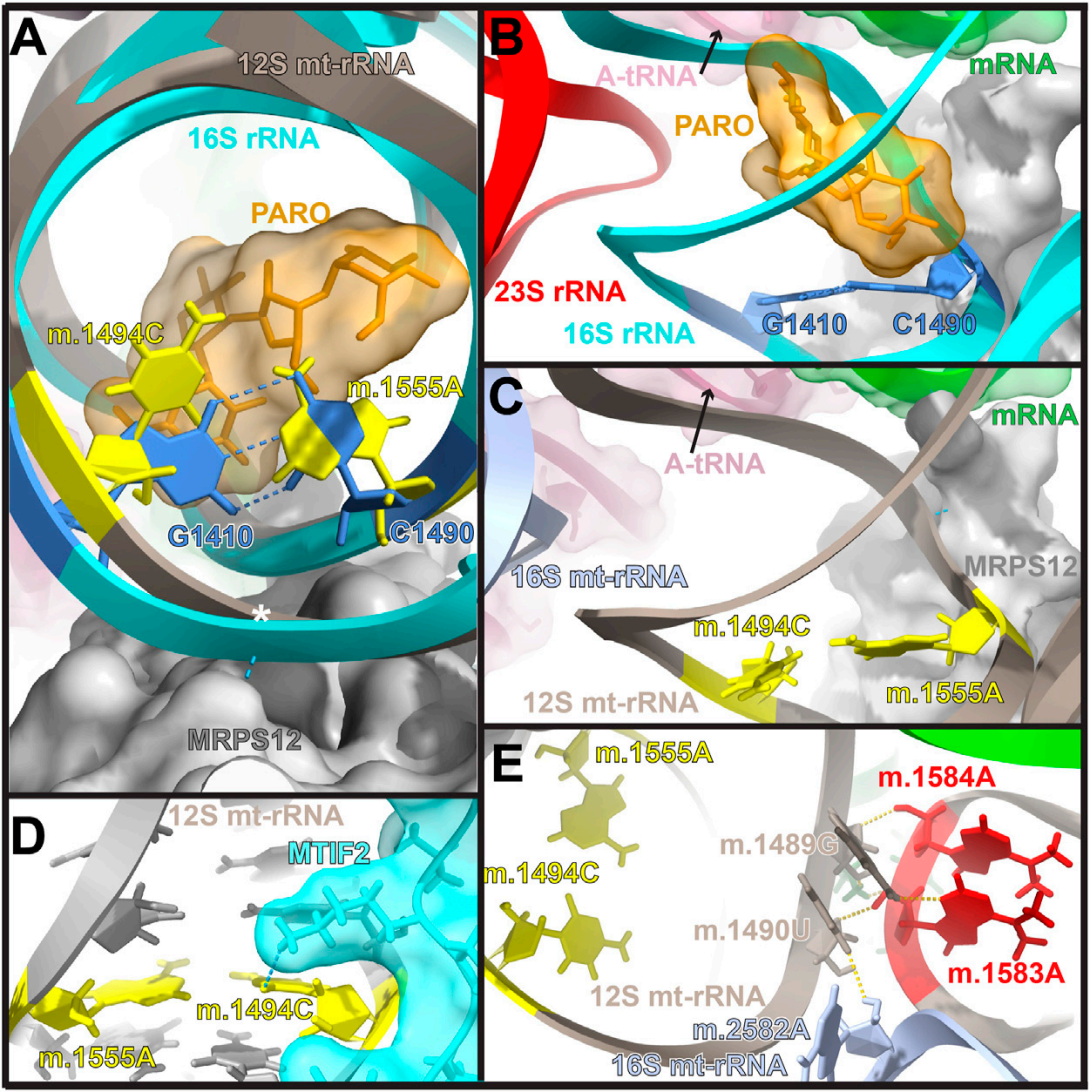
Location of residues 847C (m.1494C) and 908A (m.1555A) in the mito-ribosomal DC. **(A)** Superposition of the mitochondrial and bacterial aminoglycoside binding sites within the ribosomal decoding center. The location of residues 847C (m.1494C) and 908A (m.1555A) (yellow) in 12S mt-rRNA and that of their bacterial counterparts, G1410 and C1490 (blue), in 16S rRNA (T. thermophilus) is shown. The aminoglycoside antibiotic paromomycin (orange), present in the bacterial structure, is shown in orange. The location of position 849U (m.1496U) is indicated with a white asterisk. The hydrogen bonds between G1410 and C1490, as well as between MRPS12 and the 12S mt-rRNA backbone at position 849U (m.1496U) are indicated by blue, broken lines. **(B, C).** Bacterial **(B)** and human mitochondrial **(C)** structures showing other important molecules present at the decoding center (SSU and LSU rRNA, A-site tRNA, mRNA, and r-proteins S12/MRPS12/uS12m) are labeled and color coded to their corresponding ribbon models. **(D)** Interaction of MTIF2 (cyan) with mito-ribosomal h44. **(E)** Location of positions m.1583A and and m.1584A (red) near the 3′end of mt-12S mt-rRNA. Methods: To create panels **(A–C)**, the 2.2-Å cryo-EM human mito-ribosomal structure (Itoh et al., 2021; Itoh et al., 2022) and the structure of the *T. thermophilus* 70S ribosome complexed with mRNA, tRNA and paromomycin (PDB Accession number: 4v51) were superposed with the Matchmaker utility of Chimera X (Pettersen et al., 2021), using protein S12 as a reference chain. The structure of the human mitochondrial 28S ribosome in complex with mitochondrial IF2 (Protein Data Bank accession code 6RW5) was used for Panel **(D)**. Hydrogen bonds were identified with the H-bonds utility of Chimera X.

Error inducing antibiotics were also key in our understanding of translational fidelity. Besides streptomycin, aminoglycoside antibiotics (AGs) have been commonly used to study ribosomal decoding. Again, our understanding on the mode of action of AGs comes from bacterial studies showing that in addition to promoting general translation inhibition, AGs strongly decrease translational fidelity (Davies et al., 1965; Davies and Davis., 1968). Kinetically, AGs increase the initial binding affinity of tRNA to the ribosomal A-site tRNA during decoding and reduce its dissociation rate from the ribosome, leading to an increased error rate during translation (Karimi and Ehrenberg., 1996; Pape et al., 2000). The AG binding site is located in the major groove of helix h44 (bacterial numbering) within the DC (Figures 1, 3A) (Davies et al., 1965; Carter et al., 2000; Lynch and Puglisi., 2001). This area of the DC adopts a different structure in bacteria and mitochondria (Figures 3A–C). In particular the base pair between bacterial positions G1410 and C1490 is replaced in mitochondria by a mismatch formed by positions 847C (m.1494C) and 908A (m.1555A). Mutations at both mitochondrial positions, namely, 847C>A (m.1494C>T) and 908A>G (m.1555A>G), are well-known causative agents of maternally inherited hearing loss (Hu et al., 1991; Prezant et al., 1993; Fischel-Ghodsian, 2005; Wang et al., 2006; Bitner-Glindzicz et al., 2009; Vandebona et al., 2009). In light of the location of these mutations within the AG binding site, the observation that individuals carrying either mutation, become hypersensitive to AG-induced ototoxicity came to no surprise (Prezant et al., 1993; Zhao et al., 2004; Rodriguez-Ballesteros et al., 2006; Wang et al., 2006; Qian and Guan., 2009; Huth et al., 2011). Notably, in the absence of AGs the ototoxic mutations are well tolerated and may go unnoticed in the absence of external stimuli (Fischel-Ghodsian, 1999; Wang et al., 2006; Bitner-Glindzicz et al., 2009; Vandebona et al., 2009). Due to the toxic effect of AGs in the presence of the ototoxic variants, a non-invasive genetic test for the clinical determination of mtDNA A1555G mutation has recently been developed to be used in babies prior to the prescription of AGs (Fan et al., 2017).

Structurally, the fact that positions 847C (m.1494C) and 908A (m.1555A) are in the close neighborhood of the key A-site residues 910A (m.1557A), 911A (m.1558A), is a strong indication of their potential influence on ribosomal decoding at the mito-ribosomal A site. Additional support for this hypothesis is provided by the existence of direct contacts between MRPS12/uS12m and the neighborhood of 847C (m.1494C) and 908A (m.1555A), including the aforementioned interaction of the protein with A-site position 910A (m.1557A) (Figure 1D) and a second contact between MRPS12/uS12m Arg 82 (restrictive in *E. coli*) and the mt-rRNA backbone at position 849U (m.1496U) (white asterisk in Figure 3A). Additionally, recent studies have shown that upon formation of the initiation complex, mitochondrial initiation factor 2 (MTIF2) interacts with h44 at position 847C (m.1494C) (Figure 3D). Hence, base changes at positions 847C (m.1494C) and 908A (m.1555A) could also affect the fidelity of translation initiation at the mito-ribosomal P site (Khawaja et al., 2020; Rudler et al., 2021).

The clear influence of the mito-ribosomal AG binding site in both A- and P-site decoding, provides a rationale to the pathogenicity of the 847C>A (m.1494C>T) and 908A>G (m.1555A>G) variants. Understanding the role of the variants at the molecular level, however, has been limited to studies showing an increased inhibition of the mutant 908A>G (m.1555A>G) mito-ribosomes in the presence of AGs, relative to their wild type (wt) counterparts (Guan et al., 1996; Guan et al., 2000). The best approximation towards ascertaining the role of the ototoxic variants has been achieved *via* evidence obtained in heterologous systems. Bacterial studies showed that mutations at neighboring residues in the AG-binding region led to increased rates of readthrough and frameshifting, two of the landmarks of low translational fidelity (Gregory and Dahlberg., 1995). In the yeast mito-ribosome, the residues equivalent to 847C (m.1494C) and 908A (m.1555A) display a base paired configuration whose disruption confers AG resistance and drastically increases translational fidelity (Weiss-Brummer and Huttenhofer., 1989; He et al., 2013). When viewed in this light, the acquisition of AG hypersensitivity in humans carrying either one of the ototoxic variants was explained by the potential association of decreased mito-ribosomal fidelity to the variant-induced formation of the 847:908 (m.1494:m.1555) base pair. Support for this hypothesis has come from experiments with bacteria carrying chimeric ribosomes. By recreating the mitochondrial environment surrounding positions 847C>A (m.1494C>T) and 908A>G (m.1555A>G) by means of bacto-mitochondrial chimeric ribosomes, it was shown that the ribosomes carrying the ototoxic mutations displayed higher rates of misreading than controls and were more prone to bind AGs, explaining the AG susceptibility observed in humans (Hobbie et al., 2008a; Hobbie et al., 2008b). Further research by the same group showed that cyclic peptide antibiotics such as capreomycin and the structurally similar compound viomycin, known to be particularly active against *Mycobacterium tuberculosis* infections, also caused mt-ototoxicity (Akbergenov et al., 2011). Not surprisingly, the ribosomal binding sites of capreomycin and viomycin overlap that of AGs (Johansen et al., 2006). Again, bacterial studies with chimeric ribosomes showed that decreased fidelity during translation was linked to the effect of these antibiotics and that both ototoxic mutations rendered ribosomes hyper-susceptible to the drugs (Akbergenov et al., 2011). The conclusions obtained with bacto-mitochondrial chimeric ribosomes were in agreement with structural studies performed with RNA fragments recreating the mito-ribosomal AG binding site (Kondo and Westhof., 2008; Qian and Guan., 2009).

Genetic studies have uncovered several nuclear encoded factors that could modify the penetrance of the 908A>G (m.1555A>G). The nature of these factors is in strong agreement with the idea that defective mt-translation and, more specifically, altered levels of mito-ribosomal fidelity are central to the onset of mt-deafness. In particular, the proteins MTO1, GTPBP3 and TRMU are tRNA processing enzymes involved in the post-transcriptional modification at position 34 (“wobble” base) of several mt-tRNAs (Bu et al., 1992; Guan et al., 1996; Li et al., 2002; Bykhovskaya et al., 2004; Guan et al.,. 2006). Modification at this position is known to restrict the conformational flexibility of the “wobble” base of tRNA and to affect its ability to decode the mRNA during translation (Suzuki and Suzuki., 2014). As a result, it has been proposed that variants in these genes act by decreasing mito-ribosomal ribosomal fidelity (O'Sullivan et al., 2015). Another example of nuclear modifier is mt-protein h-mtTFB1. This protein is engaged in both mt-protein h-mtTFB1. This protein is engaged in both mt-transcription and in the post-transcriptional dimethylation of residues m.1583A and m.1584A near the 3′end of mt-12S rRNA (Figure 3E) (Bonawitz et al., 2006). Positions m.1583A and m.1584A are nearly universally conserved (Cannone et al., 2002), as is their post-transcriptional dimethylation resulting in N6,N6-dimethyladenosine, which has been observed in all domains of life with few exceptions at the species level (Xu et al., 2008; O'Farrell et al., 2004). While the mode of action of h-mtTFB1 as a nuclear modifier of the 908A>G (m.1555A>G) remains highly controversial (O'Sullivan et al., 2015; Raimundo et al., 2012; Lee et al., 2015; Cotney et al., 2009; McKay et al., 2015; Zhao et al., 2020), the idea that dimethylation of m.1583A and m.1584A might affect mito-ribosomal decoding and, more specifically, mito-ribosomal fidelity is in good agreement with bacterial data. The lack of methylation at the bacterial equivalents of m.1583A and m.1584A causes structural distortion in the packing of helices 44 and 45 near the ribosome decoding domain (Vila-Sanjurjo and Dahlberg., 2001; Demirci et al., 2010), resulting in ribosomes with altered accuracy (van Buul et al., 1984).

In summary, the available evidence strongly suggests that both ototoxic mutations, 847C>A (m.1494C>T) and 908A>G (m.1555A>G), result in an error-prone mito-ribosome. Despite these results, the link between mito-ribosomal fidelity and the ototoxic mutations has not yet been directly addressed in human mitochondria. Additionally, this evidence points to an important and insufficiently understood role for altered mito-ribosomal fidelity in mtDNA disease in humans. An important question is then, how defective mito-ribosomal translation can lead to disease.

## Translational fidelity, proteostasis, and longevity: The fidelity-longevity axis

According to Harman’s “Free Radical Hypothesis,” aging results from random deleterious events rather than being programmed, with self-inflicted oxidative damage as the primary cause behind the stochastic degeneration of organisms (HARMAN, 1956; Nystrom, 2002a; Nystrom, 2002b). A prediction of this hypothesis is that higher rates of respiration must result in greater oxidation damage and shorter lifespans (Ballesteros et al., 2001). Since mitochondrial respiration is the major source of free radicals, it comes to no surprise that these organelles stand on top of the list of culprits bearing responsibility for the ageing process (Beckman and Ames., 1998). Thus, the “Free Radical Hypothesis” is closely intertwined with the so-called “Mitochondrial Theory of Aging,” to the point that the actual question being asked is which of the two is the cause and which the consequence of aging (Wallace, 2010; Will et al., 2019). Despite the existence of ample experimental evidence supporting the “Free Radical Hypothesis”, the actual cause of the increased oxidation of macromolecules during aging was not immediately revealed by the data (Nystrom, 2003). Soon after the aforementioned studies of (Lipsett et al., 1960), Orgel first theoretically suggested that decreased levels of fidelity in the transfer of genetic information may contribute to aging. In his view, mistranslation would create a feedback loop which was responsible for an irreversible and exponential increase in error levels during macromolecular synthesis, eventually leading to “error catastrophe” (ORGEL, 1963). While initially appealing, the existence of the proposed feedback loop as an essential element of the “error catastrophe” hypothesis became a matter of intense debate (Holliday, 1996; Gallant et al., 1997). Eventually, evidence obtained in senescent bacteria completely ruled out the existence of the feedback loop, while providing an alternative hypothesis that still maintained the link between translational accuracy and aging (Dukan et al., 2000; Ballesteros et al., 2001). In this alternative view, increased accumulation of aberrant and misfolded proteins due to decreased translational fidelity in senescent bacteria, would provide the bulk substrate for oxidative attack (Dukan et al., 2000; Ballesteros et al., 2001). According to the authors, misfolded proteins would be more prone to oxidation than properly folded proteins, causing the elevated levels of oxidized macromolecules observed in aging organisms (Dukan et al., 2000; Ballesteros et al., 2001). In agreement with this idea, they found lower levels of stationary phase chaperones in cells harboring hyper accurate ribosomes, relative to wild type cells (Dukan et al., 2000; Ballesteros et al., 2001). In this light, decreased translational fidelity during senescence would provide a mechanistic explanation for the increased oxidation of macromolecules observed during aging (Nystrom, 2002a; Nystrom, 2002b; Nystrom, 2003). These results also established proteome homeostasis, or proteostasis as the primary cellular target of defective translational accuracy.

Evidence to expand the bacterial results to eukaryotes has been slowly accumulating. The seminal paper by Drummond et al. (Drummond and Wilke., 2008) studied the link between proteostasis and mistranslation, demonstrating that, across the phylogenetic spectrum, natural selection acts directly on translational accuracy *via* the selective pressure created by the toxic effect of misfolded proteins. This paper established the genetic framework to link the translational accuracy-proteostasis axis to fitness, providing a rationale to a plethora of studies on more specific aspects of this link. In clear agreement with the bacterial results of Nystrom and coworkers linking translational fidelity and proteostasis (Dukan et al., 2000; Ballesteros et al., 2001), evidence was obtained in yeast demonstrating the existence of a negative interaction between decreased fidelity and the capacity of aged cells to maintain proteostasis (von der Haar et al., 2017). Specifically, the authors pointed out to the existence of an overwhelmed chaperone system in aged cells, due to increased chemical damage and lower translational accuracy (von der Haar et al., 2017). They additionally found that reduced translational accuracy accelerated the loss of viability in this system (von der Haar et al., 2017). Furthermore, Hekman and coworkers showed that a mutation in eukaryotic translation elongation factor 2 (eEF2), that had been linked in humans to neurodegenerative diseases, produced a *ram* phenotype and resulted in the disruption of the proteostatic capacity in yeast (Hekman et al., 2012). Other studies focused on a defective form of cytoplasmic alanyl-tRNA synthetase (AlaRS) that increased the levels of mischarged transfer RNAs (tRNAs), leading to the intracellular accumulation of misfolded proteins in neurons and cardiomyocytes (Lee et al., 2006; Liu et al., 2014). Notably, defective versions of the mitochondrial equivalent of AlaRS, mtAlaRS, are a known cause of embryonic lethality in mice (Hilander et al., 2018).

The idea that loss of proteostasis, is related to aging in higher organisms has been discussed in several papers (Ben-Zvi et al., 2009; Taylor and Dillin., 2011; Lopez-Otin et al., 2013) In fact, the potential existence of a translational fidelity-proteostasis-longevity axis would provide a rationale to certain observations, such as the fact that primary fibroblasts from the extremely longevous naked mole-rats display a fourfold lower rate of amino acid misincorporation than primary mouse fibroblasts (Azpurua et al., 2013) or the strong correlation between the frequency of amino acid misincorporation and maximum lifespan in rodents (Ke et al., 2017). 

The link between the accuracy of cytoplasmic protein synthesis and longevity was explored by Xie et al. in the nematode Caenorhabditis *elegans* (Xie et al., 2019). By deleting the worm gene eukaryotic elongation factor 2 kinase (eEF2K), which promotes the accuracy of cytoplasmic protein synthesis by phosphorylating and decreasing the activity of eEF2, the authors demonstrated the acquisition of shortened lifespan in the worm *Caenorhabditis elegans* (Xie et al., 2019). The same authors showed that decreasing fidelity by inducing inadequate levels of aminoacyl-tRNA synthetases also resulted in shortened lifespans in *C. elegans* and *Drosophila* (Xie et al., 2019). The link between the accuracy of cytoplasmic protein synthesis and eukaryotes leads to proteostasis collapse (Stein et al., 2022), further underscores the toxicity of aberrant protein synthesis and its relation to aging. The fact that ribosomal pausing promotes frameshifting, stop codon readthrough, and other non-standard translational events (Precup and Parker., 1987; Qian et al., 1998; Farabaugh and Bjork., 1999; Buchan and Stansfield., 2007; Seidman et al., 2011; Manjunath et al., 2019), again links ribosomal fidelity to maintenance of proteostasis. To balance these results obtained with systems displaying lower translational fidelity, a recent study performed in parallel in yeast, *C. elegans*, and *Drosophila*, showed that a hyperaccurate mutation in ribosomal protein RPS23 (homolog of the bacterial S12) resulted in longer lifespans in the three systems (Martinez-Miguel et al., 2021).

All the evidence presented so far is in agreement with the idea that defective translational accuracy during cytoplasmic protein synthesis in eukaryotes affects proteostasis, leading to altered lifespans or, in other words, with the existence of a translational fidelity-proteostasis-longevity axis. The results of Schosserer et al. (Schosserer et al., 2015) somewhat contradicted this view by showing that decreased translational accuracy levels, due to the deletion of the conserved RNA methyltransferase NSUN5, resulted in increased the lifespan and stress resistance in yeast. The validity of Schosserer et al.´s conclusions, from the point of view of the link between mistranslation and longevity, has been questioned due to the fact that the deletion of NSUN5 induced a special form of translationally regulated stress response which, by itself, might be solely responsible for the prolonged lifespan observed (von der Haar et al., 2017; Schosserer et al., 2015). In agreement with this, cases of beneficial mistranslation under particular stress conditions have been documented both in bacteria and eukaryotes (Netzer et al., 2009; Samhita et al., 2020). While we will not dwell further on the issue of how mistranslation may become beneficial under certain stresses (for an excellent review see Mohler and Ibba., 2017), the link between the stress response and the final phenotypic manifestation of mistranslation has important bearing on what follows.

## The fidelity-proteostasis-longevity axis in regard to mitochondrial translation

Given the apparently universal conservation of the decoding mechanism, one would expect that a “fidelity toggle” similar to the one described in the bacterial ribosome exists in mito-ribosomes. *In vitro* studies with mito-ribosomes have indeed shown an increased inhibition of the mutant mito-ribosomes in the presence of AGs, relative to their wild type (wt) counterparts (Guan et al., 2000). However, no evidence of the existence of the “toggle” in the organelle was available until very recently. The existence of a potential link between the accuracy of mitochondrial translation and fitness has been difficult to prove, mostly due to the intrinsic difficulties related to the measurement of translational accuracy in the organelle. Despite this, Holbrook & Menninger showed that the accuracy inducing antibiotic erythromycin elicited a 27% increase in the lifespan of yeast cells (Holbrook and Menninger., 2002). The effect must have been mitochondrial, as petite mutants with no detectable mtDNA were not affected by erythromycin (Holbrook and Menninger., 2002). However, addressing the hypothesis of the existence of a fidelity-proteostasis-longevity axis involving mitochondrial translation required the creation of mutants carrying *ram* and restrictive base changes in their mito-ribosomes. In yeast, the ram P50R mutation in MRPS12/uS12m led to drastic effects on growth on non-fermentable carbon sources, i.e., when mitochondrial activity is required for growth (Suhm et al., 2018). Yeast strains carrying the hyper-accurate K71T MRPS12/uS12m mutation also displayed impaired mitochondrial respiration and reduced protein synthesis (Suhm et al., 2018; Ferreira et al., 2019). However, while the *ram* P50R mutant displayed aminoglycoside hypersensitivity, the K71T mutant was resistant to this effect (Suhm et al., 2018). Moreover, the lifespan of the P50R mutant was shorter than wild type, whereas the hyperaccurate K71T strain displayed an extended lifespan (Suhm et al., 2018). In agreement with the lifespan measurements, the hyperaccurate strain significantly repressed the accumulation of cellular reactive oxygen species (ROS) during aging and managed protein aggregates in a wild type fashion, whereas its *ram* counterpart displayed higher ROS levels and could not properly control protein aggregates (Suhm et al., 2018). Yeast cells carrying the error prone, but not the hyper-accurate mutation, displayed a Msn2/4-driven stress response, likely responsible for their shorter lifespan (Richman et al., 2021). The authors concluded that changes in the fidelity of mitochondrial translation modulate cytoplasmic proteostasis, with the hyperaccurate mutant repressing genes required for mitochondrial homeostasis, likely due to increased overall mitochondrial fitness. All these results place the accuracy of mitochondrial translation as an important determinant for chronological aging in yeast, in agreement with the results obtained with fidelity mutations affecting bacterial and eukaryotic ribosomes.

Despite these clear-cut results in yeast, evidence collected in metazoans has been much more difficult to interpret. Harrison & Holliday observed that the error-inducing antibiotic streptomycin caused an important decrease of life expectancy in *Drosophila* (HARRISON and HOLLIDAY., 1967), a result that has been interpreted as a direct effect of the antibiotic on mitochondrial translation (Holliday, 2005). The *Drosophila tko* ^*25t*^ (technical knockout) phenotype, caused by a L85H substitution in MRPS12/uS12m is a model of mitochondrial disease which has been proposed to be analogous to human mitochondrial deafness (Engel and Wu., 1994; Toivonen et al., 2001). However, when the equivalent alteration was introduced in *E. coli* (L56H), it was found that the mutation caused impaired assembly of S12 into bacterial ribosomes instead of a fidelity phenotype (Toivonen et al., 1999). Consistent wih this idea, the authors found decreased levels of 12S mt-rRNA and deficiency in the activities of complexes I, III, and IV, in agreement with an assembly defect (Toivonen et al., 2001). Moreover, when the *ram* MRPS12/uS12m Q116K mutation, predicted to reduce the stringency of mitochondrial translation, was introduced in *Drosophila*, a female sterility phenotype was observed that otherwise lacked any of the phenotypic features of the *tko* ^*25t*^ mutants (Gregory et al., 2001; Toivonen et al., 2001). According to the authors, these data argued against the existence of a fidelity phenotype in the case of the *tko* ^*25t*^ mutation, despite its phenotypic resemblance to the 908A>G (m.1555A>G) mutation in 12S mt-rRNA. More recent evidence suggests that the effect *tko* ^*25t*^ might be related to the cellular response to mitochondrial stress and, perhaps, to the mitochondrial unfolded protein response (mtUPR). MtUPR is a response to proteotoxic stress that is specific to mitochondria and highly sensitive to mito-nuclear protein imbalance, *i.e.*, the stoichiometric imbalance between nDNA- and mtDNA-encoded OXPHOS subunits (Jovaisaite et al., 2014). Induction of mtUPR culminates in the inhibition of mitochondrial translation (Munch and Harper., 2016; Pareek et al., 2018). While direct evidence in favor of this hypothesis is lacking in *Drosophila*, mito-nuclear protein imbalance resulting from the depletion of mito-ribosomal protein MRPS5/uS5m (and other MRPs) in *C. elegans*, gave rise to a phenotype characterized by a longer lifespan, reduced mitochondrial respiration, and an activated mtUPR pathway (Houtkooper et al., 2013). In fact, the authors found that inhibiting either mitochondrial transcription or translation resulted in similar phenotypes, leading them to claim that mito-nuclear protein imbalance may be a common underlying mechanism linking basic mitochondrial function to lifespan regulation through mtUPR activation (Houtkooper et al., 2013). Notably, increased lifespan and mtUPR were only induced when MRP knockdown was carried out during development but not when it was performed with adult *C. elegans*, as seen in other studies with long-lived mitochondrial mutants (Dillin et al., 2002; Houtkooper et al., 2013). The results of (Houtkooper et al., 2013) were called into question by (Bennett et al., 2014), by claiming that activation of the mtUPR did not strongly correlate longevity in worms. Despite these discrepancies, the finding that inhibiting translation in *C. elegans* resulted in mito-nuclear imbalance (Houtkooper et al., 2013), lends support to the idea that decreased levels of 12S mt-rRNA, due to the *tko* ^*25t*^ mutation (Toivonen et al., 2001), could lead to mito-nuclear protein imbalance and possibly elicit mtUPR. This attractive idea must remain hypothetical until proper evidence is raised.

The effect of mito-ribosomal fidelity at the organismal level has been recently addressed in mammals by the introduction of error-prone and hyper-accurate mutations in mito-ribosomes. When a *ram* mutation in protein MRPS5/uS5m (V336Y) was introduced in a human cell line, it conferred mito-ribosomal misreading in an in organello translation system (Akbergenov et al., 2018). Homozygous, knock-in mutant *Mrps5* ^V338Y/V338Y^ mice carrying the equivalent mutation were generated, leading to impaired mitochondrial function in post-mitotic cells *in vivo* and heightened susceptibility to noise trauma (Akbergenov et al., 2018). Additionally, the authors observed the coordinated up-regulation of cytosolic ribosomal proteins, an effect that had also been seen as a manifestation of 908A>G (m.1555A>G) mutation in 12S mt-rRNA and that was interpreted as a compensatory mechanism for the mitochondrial dysfunction induced by the ram mutation (Bykhoations would be tolerated up to a certain accuvskaya et al., 2009; Akbergenov et al., 2018). In the case of the 908A>G (m.1555A>G) mutation in 12S mt-rRNA, Myc/Max pathway genes were also up-regulated, possibly explaining the increase in the levels of cytoplasmic ribosomal genes (Bykhovskaya et al., 2009). According to Akbergenov et al. (Akbergenov et al., 2018) this phenotypical coincidence could be explained by the fact that stochastic misreading, caused by ram mutations, would be tolerated up to a certain accuracy threshold which, these mutations by themselves do not surpass.

Ferreira et al. (2019) also generated mammalian models of defective mito-ribosomal fidelity by raising mice strains carrying error-prone and hyper-accurate mutations in MRPS12/uS12m. Homozygous young mice with a ram mutation at position K72I of MRPS12/uS12m displayed a dramatic reduction of the rate of mitochondrial translation, both in liver and heart mitochondria (Ferreira et al., 2019). In agreement with the ram nature of the K72I mutation, the translation defect observed in MRPS12/uS12m^K72I/K72I^ mice was amplified by AGs (Ferreira et al., 2019; Richman et al., 2021). These translation defects were corrected with age in MRPS12/uS12m^K72I/K72I^ mice due to a compensatory response caused by elevated mitochondrial biogenesis, higher cellular proliferation in the liver, and the activation of the mitochondrial stress response (Ferreira et al., 2019). In summary, while the two studies involving mice carrying ram mito-ribosomal mutations are not directly comparable, due to the different assays performed, they both reach the overall conclusion that these mutations are consistent with viable, almost normal mice, at least in the absence of toxic external factors, a phenotype not too different from that of the 908A>G (m.1555A>G) mutation in 12S mt-rRNA (Hu et al., 1991; Prezant et al., 1993; Guan et al., 2000; Hobbie et al., 2008a; Hobbie et al., 2008b; Akbergenov et al., 2011; Akbergenov et al., 2018; Ferreira et al., 2019).

In regard to the distinct phenotypes displayed by ram mitochondrial mutants in yeast and animals (*C. elegans* and mammals), the authors underscored the importance of the different types of stress responses activated in the two systems to deal with mitochondrial mistranslation. Accordingly, activation of a mitochondrial specific stress response in animals, instead of the general Msn2/4-driven stress response of yeast, allowed a more sophisticated reaction to mitochondrial mistranslation in the former that, with time, compensated the deleterious effects of mistranslation largely by inducing mitochondrial biogenesis (Suhm et al., 2018; Ferreira et al., 2019; Richman et al., 2021).

A hyper-accurate model of mitochondrial translation was generated by introducing the K71T mutation in MRPS12, is conserved between yeast, worms and mice (Ferreira et al., 2019; Richman et al., 2021). Initial experiments showed that, in contrast to mice with ram mito-ribosomes, hyper-accurate MRPS12/uS12m^K71T/K71T^ mice displayed a slight reduction in mitochondrial protein synthesis (Ferreira et al., 2019). The fact that mitochondrial translation in hyper-accurate mice was much more insensitive to AG-induced inhibition than that of their error-prone counterparts, was taken as confirmation of the hyper-accurate phenotype (Ferreira et al., 2019). However, unlike error-prone mice, mitochondrial specific stress response pathways were not activated in hyper-accurate mice to rescue the protein synthesis defect, leading to compromised OXPHOS function and to cardiomyopathy in aged individuals (Ferreira et al., 2019). To explain the physiological consequences of the hyper-accurate mutation, the authors argued that in mice mitochondrial translation rate must be more important than translational accuracy, in regard to the ultimate phenotypic manifestation of fidelity mito-ribosomal mutations.

Further studies with the above mouse models of mito-ribosomal fidelity finally provided a glimpse of the phenotypic complexity underlying the manifestation of these type of mutations. Shcherbakov and coworkers studied the effects of decreased mito-ribosomal fidelity in two types of post-mitotic tissue with high energy demand, brain and skeleton muscle (Scherbakov et al., 2020; Shcherbakov et al., 2021). Surprisingly, they found an age-dependent, disparate response in either tissue. While they observed accelerated metabolic aging, lipid accumulation, and increased inflammation in skeleton muscle, a mito-hormetic response was induced in the brain that mitigated the age-associated decline in mitochondrial gene expression (Scherbakov et al., 2020; Shcherbakov et al., 2021). Richman and coworkers also tested the tissue-dependent manifestation of mito-ribosomal mistranslation, but added a new level of complexity by including diet as a variable in their experiments. For example, when tested in heart tissue, a high-fat diet (HFD), stimulated mitochondrial translation in both error-prone (MRPS12/uS12m^K72I/K72I^) and hyper-accurate (MRPS12/uS12m^K71T/K71T^) mice, while sustaining the stability of the newly synthesized proteins only in the latter (Ferreira et al., 2019; Richman et al., 2021). In the liver, however, HFD reduced both mitochondrial translation and the stability of newly synthesized proteins in the error-prone, but not in the hyper-accurate mice (Richman et al., 2021). The authors noted the existence of tissue-specific cellular responses to altered mitochondrial translation, leading both to cardiac defects and liver protection in hyper-accurate, HFD-fed mice and to the absence of cardiac defects, but lipid accumulation and impaired liver function, in their error-prone counterparts (Richman et al., 2021). In contrast to HFD, a normal chow diet (NCD) reduced mitochondrial translation in both types of mice, but led to shortened lifespans in hyper-accurate, but not error-prone mice (Ferreira et al., 2019; Richman et al., 2021). Parallel assays performed in *C. elegans* were consistent with the observed mice lifespans (Richman et al., 2021). According to the authors, worms carrying either fidelity mutation induced the mtUPR response, as judged by the increased levels of hsp-6 (Richman et al., 2021). The response that was somewhat larger in the hyper-accurate mutants, albeit resulting in shorter lifespans relative to their error-prone counterparts (Ferreira et al., 2019; Richman et al., 2021).

While these recent investigations with mitochondrial models of translational fidelity provide an unprecedented view on the complexity underlying of phenotypic manifestation of altered mistranslation in the organelle, the current picture is far from complete and plagued with yet unresolved discrepancies. To resolve these discrepancies and to understand the extent of damage caused by mito-ribosomal mistranslation at the molecular level, it is important that new studies make use of model systems harboring mito-ribosomal mutations to directly address the measurement of translation rates, mito-ribosomal fidelity, and mitochondrial protein misfolding. Moreover, the disparate phenotypes observed in yeast and animals, as well as in different animal tissues, require further investigation to fully elucidate how a given level of molecular damage can trigger different stress responses in different cellular systems and to learn how these responses influence the final phenotypic manifestation of mitochondrial mistranslation. Regarding this phenotypic manifestation, the fact that both the accuracy and the translation rates elicited by mito-ribosomal fidelity mutations are important, as reported in mice (Ferreira et al., 2019; Richman et al., 2021), suggests that mitochondrial ribosome stalling and its associated-rescue mechanisms might play a role. For example, it is possible that the increased ribosome pausing observed in aging (Stein et al., 2022) might also affect mitochondrial translation, leading both to mistranslation and increased activation of mitochondrial RQC. Future work targeting mitochondrial RQC factors could be used to learn about their role in ensuring mito-ribosomal fidelity. Finally, the observation that the activation of the mtUPR response in *C. elegans* with hyper-accurate mito-ribosomes resulted in reduced longevity (Richman et al., 2021), is in stark contrast to the results of Houtkooper et al. (Houtkooper et al., 2013) claiming a positive correlation between the level of mtUPR and lifespan. Perhaps, these conflicting results could be explained by taking into consideration some key points raised by Bennet et al. regarding the activation of the mtUPR in *C. elegans* (Bennett et al., 2014). First, we have already mentioned Bennet et al.´s concern with the claim that mtUPR and longevity are strongly correlated in worms, as their research disproved the existence of such correlation (Bennett et al., 2014). Second, the exclusive use of single mtUPR reporters, such as hsp-6, as proof of the activation of mtUPR has been called into question by these authors (Bennett et al., 2014). All these issues must be addressed by future work to achieve a clear picture of how mitochondrial mistranslation leads to disease. In the meantime, and simplistically considering that all ram mito-ribosomal mutations are likely to elicit the same phenotypic manifestation, one could tentatively argue that these mutations should become highly toxic in the ear, possibly under the influence of external stimuli, as illustrated by the clinical manifestations of the two ototoxic 12S mt-rRNA mutations and the V338Y mutation in MRPS5/uS5m (mouse numbering) (Hu et al., 1991; Prezant et al., 1993; Guan et al., 2000; Hobbie et al., 2008a; Hobbie et al., 2008b; Akbergenov et al., 2011; Akbergenov et al., 2018; Ferreira et al., 2019).

## Are there more pathogenic mito-ribosomal fidelity mutations?

There are multiple reasons to believe that there might exist many yet unidentified pathogenic mutations affecting mito-ribosomal fidelity. First, the evidence presented in the previous sections suggests that such mutations will probably display very subtle phenotypes, possibly remaining unnoticed in the absence of external toxic agents, or perhaps playing a role in late onset diseases such as neurodegeneration. Second, fidelity mutations are quite abundant, as demonstrated by bacterial studies showing that defective ribosomal fidelity can be associated to mutations mapping to many regions of the two ribosomal subunits, affecting both their protein and RNA components (Sun et al., 2011; Tran et al., 2011; O'Connor., 2007; Liiv and O'Connor., 2006; O’Connor and Dahlberg., 1995; ; McClory et al., 2010). Third, many potentially pathogenic mt-rRNA variants have been reported. Our group has used comparative methods to study variants mapping to conserved positions of mt-rRNA, leading to their scoring as potentially pathogenic, based on their degree of disruption of mito-ribosomal structure and function (Smith et al., 2014; Elson et al., 2015a; Elson et al., 2015b; Aw et al., 2018; Haumann et al., 2020). In addition to these variants, there exists a much larger group of moderately-to-non-conserved mt-rRNA variants that have been associated with disease and whose disruptive potential is unassessable by comparative methods (Smith et al., 2014; Vila-Sanjurjo et al., 2021). Strikingly, the two ototoxic mutations, 847C>A (m.1494C>T) and 908A>G (m.1555A>G), would fall into the unassessable set of variants (Smith et al., 2014). Hence, the possibility that other pathogenic mutations affecting mito-ribosomal fidelity exist is quite high.

The recent availability of a near-atomic cryo-EM human mito-ribosomal structure at 2.2-Å resolution (Itoh et al., 2021; Itoh et al., 2022), has prompted us to study the potential disruptive role of a group of mt-rRNA variants claimed to be involved in mitochondrial deafness (Vila-Sanjurjo et al., 2023). While up to now, only the two ototoxic mutations, 847C>A (m.1494C>T) and and 908A>G (m.1555A>G) of human 12S mt-rRNA, have been unambiguously established as causes of mitochondrial deafness, there exists an important number of studies with patients in which deafness was possibly associated with the presence of variants mapping to the mt-rRNA genes. In total, we analyzed 93 of such deafness-associated variants mapping to the mt-SSU (83) and mt-LSU (10), originating from 35 studies reported in the literature and/or MITOMAP (Ruiz-Pesini et al., 2007). Our structural analysis led to the conclusion that more than half of them, 49, have the potential to cause structural distortions that could lead to defective mito-ribosomal function (Vila-Sanjurjo et al., 2023). We were particularly surprised by the number of variants mapping to the vicinity of protein MRPS12/uS12m, a total of 11, with 10 of them deemed potentially disruptive (Vila-Sanjurjo et al., 2023). Given the high structural homology between the *E. coli* and mitochondrial versions of protein S12 (Figure 2), our results could be tentatively interpreted as further proof of the involvement of mito-ribosomal fidelity in the etiology of mt-rRNA-induced deafness. Ample potential for the possibility of additional 12S rRNA deafness-inducing variants among the cohort studied exists, due to the previously noted abundance of fidelity phenotypes among rRNA mutants. Unfortunately, structural analysis alone cannot be used to unambiguously establish the pathogenicity of these variants. Hence, understanding the mode of action of mt-rRNA mutations can be considered one of the last unexplored frontiers in our understanding of the role of mtDNA mutations in disease (Vila-Sanjurjo et al., 2021).

## Conclusion

The high-resolution structures of mito-ribosomes clearly show that their DC is almost identical to those observed in other domains of life. Therefore, it is expected that genetic decoding proceeds in mitochondria in a fashion similar to what has been described in bacterial systems. Hence, the pathogenic effect of the known mito-ribosomal mutations affecting the functioning of the mitochondrial DC is most likely explained by defective genetic decoding affecting translational fidelity. The results obtained with mito-ribosomal fidelity mutations in the last few years provided proof of the existence of a fidelity toggle in the organelle and offered a first glimpse of how the this toggle is exquisitely tuned, to a particular level of accuracy. Hence, the toggle constitutes yet another example of the conservation of translation systems.

The emergence of the first models of mito-ribosomal fidelity have, for the first time, allowed us to explore its role in mitochondrial disease and longevity. While the first reports have confirmed the original suspicion of the existence of a crucial role for mito-ribosomal fidelity in ensuring proper mitochondrial function, they have also underscored the complexity underlying its phenotypic manifestation. A translational fidelity-proteostasis axis, which becomes a translational fidelity-proteostasis-longevity in eukaryotes, apparently exists in all the organisms so far tested. While the phenotypical effects of this axis are conspicuously expressed through the action of bacterial, cytoplasmic, and yeast mitochondrial translation systems, they could remain buffered in the case of metazoan mitochondrial translation systems by the existence of additional layers of regulatory complexity. These layers include the existence of more complex stress response systems in metazoans (particularly in mammals), tissue-specific regulation of gene expression, and the effect of diet (Aw et al., 2018; Ferreira et al., 2019; Scherbakov et al., 2020; Richman et al., 2021; Shcherbakov et al., 2021). Hence it should come as no surprise that yeast and mice displayed disparate phenotypes as a result of fidelity mutations (Suhm et al., 2018; Ferreira et al., 2019; Richman et al., 2021). The fact that both mice and *C. elegans* launched a mitochondrial-specific stress response, whereas yeast could only launch a general stress response was likely behind these disparities (Suhm et al., 2018; Richman et al., 2021). While these studies provide an unprecedented view on the intricacies of the phenotypic manifestation of defective mito-ribosomal fidelity, much work is still needed to gain a converging view of how defects at the level of mitochondrial translation result in the complex phenotypic manifestations leading to disease.

## Footnotes

1
*Gene and parenthesized genomic positions, the latter preceded by the sufix“m.”, are provided for mt-rRNA residues.*
